# Flexible all-polymer waveguide for low threshold amplified spontaneous emission

**DOI:** 10.1038/srep34565

**Published:** 2016-09-30

**Authors:** José R. Castro Smirnov, Qi Zhang, Reinhold Wannemacher, Longfei Wu, Santiago Casado, Ruidong Xia, Isabel Rodriguez, Juan Cabanillas-González

**Affiliations:** 1 Madrid Institute for Advanced Studies in Nanoscience, IMDEA Nanociencia, Calle Faraday 9, Ciudad Universitaria de, Cantoblanco, 28049, Spain; 2 Key Laboratory for Organic Electronics and Information Displays & Institute of Advanced Materials, National Jiangsu Synergistic Innovation Center for Advanced Materials (SICAM), Nanjing University of Posts and Telecommunications, 9 Wenyuan Road, Nanjing 210046, P.R. China

## Abstract

The fabrication of all polymer optical waveguides, based on a highly fluorescent conjugated polymer (CP) poly(9,9-dioctylfluorene-alt-benzothiadiazole) (F8BT) and a mechanically flexible and biodegradable polymer, cellulose acetate (CA), is reported. The replication by hot embossing of patterned surfaces in CA substrates, onto which high quality F8BT films can be easily processed by spin coating, is exploited to produce an entirely plastic device that exhibits low optical loss and low threshold for amplified spontaneous emission (ASE). As a result, highly transparent and flexible waveguides are obtained, with excellent optical properties that remain unaltered after bending, allowing them to be adapted in various flexible photonic devices.

The study of CPs has attracted a lot of attention over the past two decades because of their remarkable electroluminescent and semiconducting properties^1^,^2^. Up to date, CPs have found applications in different areas like organic light-emitting diodes^3^, organic field-effect transistors^4^, organic solar cells^5^, and memory devices^6^, among others. An additional attractive property of CPs is their compatibility^7^,^8^,^9^ with a considerable variety of organic and inorganic materials, permitting for instance the use of mechanically flexible substrates^10^,^11^,^12^. One of the distinct advantages of stretchable and bendable systems over rigid ones is the fact that they can be adapted and adhered to curved surfaces, which allows to broaden the range of applications areas. Apart from flexibility, another advantage of CPs is their simple processability into partially transparent thin films. Since it was first reported by Moses *et al*.^13^, a new exciting area of research has been the study of lasing^14^ and Amplified Spontaneous Emission (ASE)^15^ in CPs. In addition, several strategies have been put in practice to obtain low thresholds. It has been already established that the chemical structure of the conjugated polymer has a considerable effect on the optical gain properties of the material^16^,^17^,^18^. Other approaches^19^,^20^ to reduce the ASE threshold emphasize on the optimization of the geometry of the optical structures, taking advantage of the simple solution-based processing of CPs to fabricate low-loss waveguides. The waveguide structure enhances the confinement of light in the optical gain layers, which boosts ASE.

In this regard, it is of a high importance the fabrication of ridge waveguides which, preserving or even surpassing the optical amplifying properties of slab waveguides, allow for their integration in a range of applications via appropriate optical design. Herein we show a simple method to build flexible all-polymer waveguides. The distinguishing feature of such route is that it can be applied to a wide range of materials to develop nano/micro structures, paving the way to the potential fabrication of many different waveguide-based systems. The proposed system is based on the highly fluorescent conjugated polymer F8BT as optical gain medium^21^,^22^ and a mechanically flexible and biodegradable CA polymer, as a substrate. In addition to layer compatibility, CA and F8BT present a significant refractive index contrast, a fact that enhances light confinement and optical amplification of the waveguides^23^,^24^. The optical properties of F8BT as a gain medium have been studied earlier both in solution and grating-coupled waveguide amplifiers^25^. Herein, we fabricate CA/F8BT and Quartz/F8BT infinite slab waveguides (CAISWG and QISWG, respectively), as well as CA/F8BT asymmetrical ridge waveguides (ARWG), the latter produced by hot embossing. Furthermore, we encapsulate the ARWGs with a solution processed CA layer, giving rise to a symmetrical ridge waveguide (SRWG). This cladding layer functions as a barrier^26^, which prevents the optical gain layer to react with oxygen and water, avoiding photo-induced oxidation and degradation, a well-known vulnerability^27^ of CPs. We model the TE_00_ mode intensity distribution in two dimensions in the ARWG and SRWG and obtain large confinement factors in the active layers. These new features lead to excellent optical amplifying performance: an ASE threshold as low as 9.3 kW/cm^2^, and a loss coefficient of 3.1 cm^−1^, remarkable values according to literature review^28^,^29^.

## Results and Discussion

**Figure 1:**
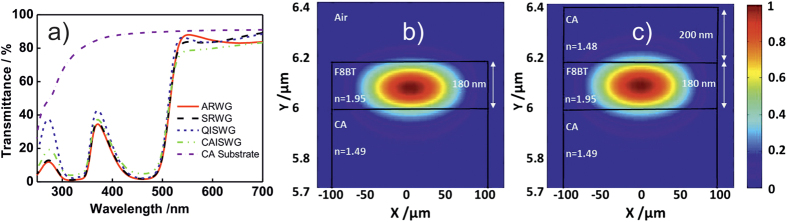
(**a**) Transmittance spectra of CAISWG (green dash dot dot line), QISWG (blue short dash), ARWG (red solid line), SRWG (black dash line) and the used CA substrate (purple short dash dot). Simulation of TE_00_ waveguide mode profiles at the wavelength of 570 nm in (**b**) ARWG and (**c**) SRWG.

The remarkable film forming properties of the proposed materials and their ease of processing over geometrically random surfaces gives rise to highly transparent samples, with transmittance values ranging between 80% and 90% in the visible spectrum where no absorption is observed from F8BT, as depicted in Fig. 1a. The microstructuring of CA substrates or the addition of encapsulating CA layers did not affect the optical quality, observing almost identical transmission spectra in all of the waveguides, and negligible differences compared to a CA film used as a reference. Complementary absorption spectra displayed in Figure S1 show a strong absorption at around 330 nm and 450 nm, directly ascribed to π-π* transitions in F8BT^30^. In order to analyze the optical confinement, a simulation of the TE_00_ mode intensity distribution was performed for the ARWG and SRWG, as displayed in Fig. 1b,c, respectively. The simulation was carried out by means of the semivectorial version of the WGMODES^31^,^32^ finite difference mode solver for dielectric waveguides. In the case of the SRWG, the geometry is more symmetric as both CA layers have similar refractive index. As a result of the larger refractive index difference between core and upper-cladding (air) in the ARWG there is a small shift of the field intensity distribution towards the substrate. The peak intensity in the SRWG is lower than in the ARWG for equal total power of the mode, because the distribution of the former is more spread out. Given that ASE is a consequence of wave propagation within an optical gain medium, confinement of the transmitted mode inside the gain layer is crucial for light amplification to take place. We calculate the fraction of light intensity propagating through the core of each waveguide. This quantity can be expressed by the confinement factor, which can be calculated for a polymer waveguide of core thickness *d* and width *w* via Eq. (1):


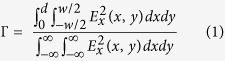


where the integral in the numerator extends over the core and x and y correspond to the lateral and thickness dimensions of the waveguide, as indicted in Fig. 1b,c. The confinement factors for the ARWG and SRWG, calculated numerically, are 0.798 and 0.773 respectively. The strong optical confinement in the F8BT layer in both geometries is suitable to achieve outstanding optical gain and ASE properties^33^. The results of complementary analytical calculations performed for QISWG and CAISWG can be found in Figure S2, from which confinement factors of 0.804 and 0.797, respectively, were extracted.

**Figure 2:**
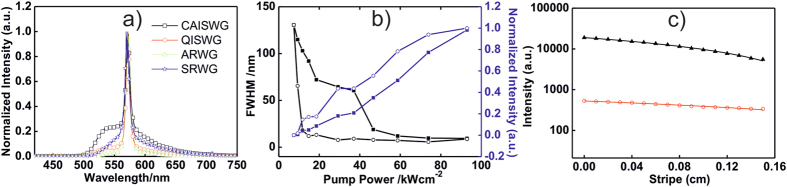
(**a**) Normalized output spectra of CAISWG (black solid line with squares), QISWG (red solid line with circles), ARWG (green solid line with triangles) and SRWG (blue solid line with stars). (**b**) Full widths at half-maximum values and their emission peak intensities of CAISWG (black and blue squares respectively) and SRWG (black and blue circles respectively), as a function of the incident laser fluence. (**c**) Dependence of the emission intensity at λ_ASE_ on the length of the unpumped region between the edge of the excitation stripe and the substrate edge of ARWGs (black triangles) and SRWGs (red circles). Black solid line and red solid line represent a linear fit of each data set.

The emission spectra of the four systems are presented in Fig. 2a. After optical pumping, all samples exhibit ASE, with peaks centered at around 570 nm. The narrowing of the spectra of the four systems after increasing the pump power can be found in Figure S3. When the pump intensity was augmented above 20 kW/cm^2^, the spectral width was reduced to 7–9 nm for all cases. This trend is distinctive of ASE processes, being attributed to spontaneously emitted photons which are amplified by stimulated emission as they propagate along the gain medium. Following standard procedures, we extract the ASE threshold value as the excitation power at which the full width at half maximum (FWHM) of the emission band is reduced to half of the photoluminescence linewidth. The evolution of the FWHM as a function of the power density for CAISWG and SRWG is shown in Fig. 2b. Analogous measurements for QISWG and ARWG can be found in Figure S4.

We perceive a strong line narrowing effect combined with a slight increase in the slope of the input-output curve. Significantly, the ASE threshold in ARWG, 18.6 kW/cm^2^, was found to be lower than the corresponding value for CAISWG, 29.3 kW/cm^2^, suggesting that the ridge waveguide configuration exhibits superior optical amplifying properties. Also, the fact that the ASE threshold of QISWG is similar to that of ARWG reveals that F8BT waveguiding properties on imprinted CA or quartz substrates are comparable. To explore the ASE behavior further, the waveguide losses were measured. For this purpose, the length of the pump stripe was maintained constant while it was gradually moved away from the sample edge. Since the stripe length is fixed, the emission from edge of the sample is reduced as a consequence of the waveguide losses. The experimental data (Fig. 2c) were then adjusted following an exponential dependence on the length of the unpumped region according to 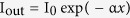, where α is the loss coefficient, which comprises both absorption and scattering losses, being I_0_ the spontaneous emission intensity. Loss coefficients of 3.1 cm^−1^ and 8.1 cm^−1^ were calculated for SRWGs and ARWGs, respectively, equivalent to approximately 60% decrease of the total loss for symmetric WGs. Loss coefficients of 10.5 cm^−1^ and 5.7 cm^−1^ were extracted from the data shown in Figure S5 for CAISWG and QISWG respectively. The factors that influence this loss in the waveguide comprise absorption, as well as scattering from surface or interface roughness and/or film inhomogeneity. In order to shed light into the different loss coefficients found for the waveguides, the surface topographies of the same were characterized by atomic force microscopy (AFM) (Figure S6). It was found that the surfaces in CAISWG and ARWG exhibited root mean square roughness (RRMS) values of 1.2 nm and 1.0 nm, respectively. The surface roughness of SRWGs was similar to that of QISWG, with RRMS values of 0.7 nm and 0.5 nm, respectively. We measured the roughness in at least 4 different regions on each sample and obtained very similar values, so that we can assume that the RRMS measurements are representative. The cause of increased roughness in CAISWG and ARWGs with respect to QISWG is likely related to surface functionalization of CA substrates. In the former, UV radiation was necessary in order to render the CA substrate more hydrophilic, making possible the F8BT film formation. This treatment is known to increase the roughness of the treated substrate. Likewise, subsequent deposition of a CA layer on top of F8BT to obtain SRWG had as result a smooth topography due to covering of surface-induced inhomogeneity associated to UV treatment. Interestingly, the samples with flatter surfaces revealed lower loss coefficients in comparison with the systems presenting rougher surfaces. This observation confirms that surface roughness is the prevailing factor that determines the loss coefficients of the waveguides. Furthermore, the optical gain coefficients at pump powers just above the ASE threshold were calculated with the variable stripe length method^34^, yielding values which fluctuate from 12 cm^−1^ to 14 cm^−1^, close to reported ones in literature^35^. The estimated threshold, FWHM, ASE wavelengths, as well as the loss and gain coefficients, and the calculated confinement factors for each of the waveguides are shown in Table 1.

**Figure 3:**
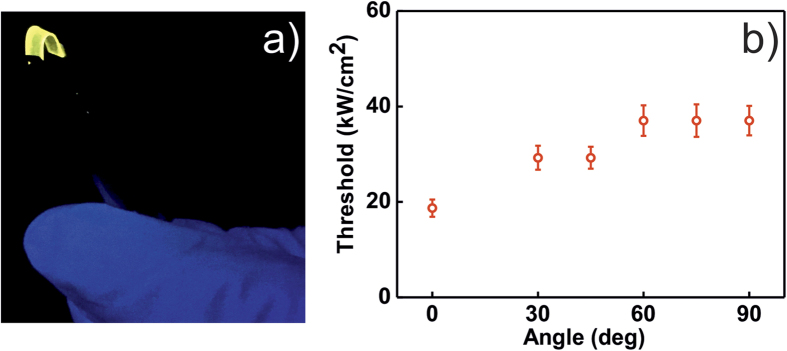
(**a**) Photograph of a bent ARWG substrate. (**b**) Variation of the ARWG threshold values with the bending angle. The measurements were carried out 5 times at each angle. Error bars represent the standard deviation of these values.

Finally, the ASE threshold values for ARWGs were obtained for different bending angles. Indeed, given that the substrates are highly flexible, they can be bent easily without apparent formation of cracks in the surface. A sample image of a bent ARWG is shown in Fig. 3a. The bending angle was defined as the angle between the tangents at the opposite substrate edges where the center of the sample is facing the optical pump beam. In Fig. 3b it can be seen that the threshold values of the sample do not suffer major variation up to a bending angle of 90°. These results prove the robustness and optical gain stability of the waveguides.

## Conclusions

In conclusion, we have developed flexible, transparent all polymer waveguides with enhanced ASE performance via hot embossing and encapsulation. A low threshold of 9.3 kW/cm^2^ and a loss coefficient of 3.1 cm^−1^ were achieved for symmetric waveguides under optical pumping. These values are comparable to those previously reported in the literature for rigid substrates. The attained efficiency is ascribed to the large confinement factors and optimized transmission loss of guided light within microstructured ridges. Furthermore, the optical properties of the polymer waveguides did not show major variation in performance upon bending. The approach proposed does not involve complex processing steps, it is low cost and can be up-scaled. Hence, these results pave the way for further developments in the field of flexible and transparent active photo-electronic devices.

## Methods

### Imprinting of waveguide microstructures

The waveguide structures were made using standard photolithographic process on a silicon substrate with a 7-μm thick silica buffer layer (n = 1.46) to fabricate master waveguide. The widths of waveguides are 5, 10, 20, 50, 100, 200 μm with the same depth of 6 μm. The Si waveguide master mold was initially treated with a fluorosilane through vapor deposition of 1H,1H,2H,2H-perfluorodecyl trichlorosilane (Alfa Aesar, MA) as release agent to reduce the surface energy, facilitating the demolding process. The geometrical design of the ridge WG that was used for the master mold can be found in the Supporting information Figure S7. Next, the waveguide ridges on the Si mold were replicated in an elastomeric polymer poly(dimethyl)siloxane (PDMS) (Sylgard 184, Dow Corning) to produce a working mold. Subsequently, using the PDMS as stamp, the WG ridges were imprinted onto a CA film (Compact Nanoimprint Tool (CNI Tool), NILT, Denmark). During the hot embossing process, substrates were heated at 180 °C with an applied pressure of 7 Bar for 1200 s. The sample was subsequently allowed to cool down to a temperature of 70 °C before the pressure was released and the imprinted substrate removed.

### Fabrication of all polymer F8BT waveguide

A flexible CA film (Refractive index n = 1.49) of 500 μm thickness with flat surfaces provided by Clarifoil (Celanase, UK) was used as a substrate. A highly fluorescent conjugated polymer poly(9,9-dioctylfluorene-alt-benzothiadiazole) (F8BT) (American Dye Source, refractive index n = 1.95 at 570 nm, Mn = 33 kg/mol, Mw = 217 kg/mol) was dissolved in toluene (Sigma Aldrich) at a concentration of 25 mg/ml and the solution was filtered through a 0.22 μm syringe filter. F8BT films were deposited onto CA films with the WG ridge structures imprinted on the surface, by spin coating (Laurell WS-400A-6TFM/LITE), giving rise to ARWGs. The F8BT thin films, spin coated at 2000 rpm for 1 minute, with the acceleration set at 12000 rpm/s, had an average thickness of 180 nm. The SRWG consisted of an ARWG with an additional CA layer spin coated onto the deposited F8BT film. This capping layer was produced by spin coating a CA solution (40 mg/ml) (Mw = 61 000, resulting refractive index of the film n = 1.48 at 570 nm, from Sigma Aldrich), in 4-hydroxy-4-methyl-2-penthanon (diacetone alcohol, Sigma Aldrich). The CA films with a thickness of 200 nm were spin coated at 3000 rpm for 1 minute, with an acceleration set at 12000 rpm/s. CAISWGs and QISWGs were prepared by spin coating F8BT films on CA and quartz respective substrates with flat surfaces, following similar concentration and rotation speeds.

### Optical and structural characterization

The set-up for measuring the ASE consisted of a Nd:YAG laser (355 nm) (TEEM Photonics) delivering pulses of 300 ps duration at 30 Hz repetition rate as pumping source. The photoluminescence arising from the edge of the waveguide was spectrally dispersed with a spectrometer (SP2500, Acton Research) equipped with a liquid nitrogen cooled back-illuminated deep depletion CCD (Spec-10:400BR, Princeton Instruments). The pumping intensity was regulated with neutral density filters. The threshold values were obtained from the fluence dependence of the FWHM and the output intensity. To obtain an optimum measurement, the input laser beam was positioned parallel to the ridges and a cylindrical lens was used for focusing. From the different ridge geometries, the wider slots yielded larger collected fluorescence intensity. Hence, all measurements reported here were subsequently taken from the 200 μm ridges. Absorbance and transmittance spectra were recorded using a Varian Cary 50 UV-VIS spectrophotometer over a spot size of 1 mm. Atomic force microscopy (AFM) images were obtained using a JPK NanoWizard II AFM working in contact mode, attached to a Nikon Eclipse Ti inverted optical microscope. Standard Olympus silicon nitride cantilevers of 0.05 N/m spring constant and 18 kHz resonant frequency were employed in air conditions. SEM characterization was performed by means of a Carl Zeiss Auriga microscope operating at 3 kV.

## Additional Information

**How to cite this article**: Smirnov, J. R. C. *et al*. Flexible all-polymer waveguide for low threshold amplified spontaneous emission. *Sci. Rep.* **6**, 34565; doi: 10.1038/srep34565 (2016).

## Supplementary Material

Supplementary Information

## Figures and Tables

**Table 1:** ASE peak wavelengths, FWHM values, ASE power density thresholds, loss and gain coefficients and calculated confinement factors (Γ) for QISWG, CAISWG, ARWG and SRWG.

Sample	ASE Peak (nm)	FWHM (nm)	Threshold (kW/cm^2^)	Loss (cm^−1^)	Gain (cm^−1^)	*Γ*
QISWG	571,6	7,1	18.6	5.7	13.4	0.804
CAISWG	571,5	9,7	29.3	10.5	14.9	0.97
ARWG	573,4	9,6	18.6	8.1	14.3	0.798
SRWG	569,1	8,9	9.3	3.1	12.2	0.773

Gain measurements were performed at a pump power density just above the threshold of each sample.
