# Supplementary material for: Flexible all-polymer waveguide for low threshold amplified spontaneous emission
Source: Sci Rep. 2016 Sep 30;6:34565. doi: 10.1038/srep34565 (PMC5043348; doi:10.1038/srep34565)
Supplement: Supplementary Information [file srep34565-s1.pdf]

Supplementary Information

Flexible all-polymer waveguide for low threshold amplified spontaneous emission

José R. Castro Smirnov[1], Qi Zhang[2], Reinhold Wannemacher[1], Longfei Wu[1], Santiago Casado[1], Ruidong Xia[2]*, Isabel Rodriguez[1] and Juan Cabanillas-Gonzalez[1]*

[1] Madrid Institute for Advanced Studies in Nanoscience, IMDEA Nanociencia, Calle Faraday 9, Ciudad Universitaria de Cantoblanco28049, Spain.
[2] Key Laboratory for Organic Electronics and Information Displays & Institute of Advanced Materials, National Jiangsu Synergistic Innovation Center for Advanced Materials (SICAM), Nanjing University of Posts and Telecommunications, 9 Wenyuan Road, Nanjing 210046, P.R., China.

*corresponding author: iamrdxia@njupt.edu.cn; juan.cabanillas@imdea.org;

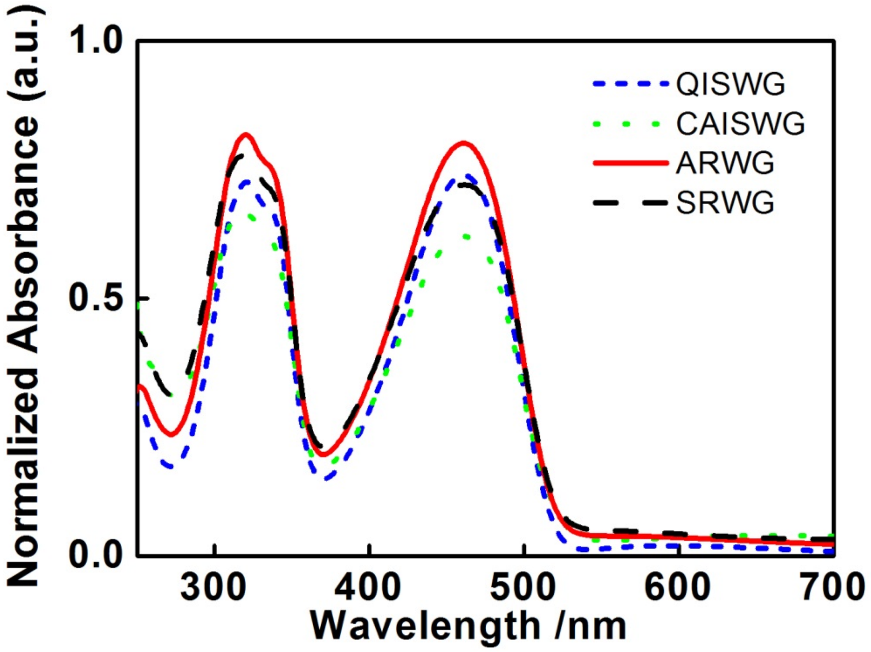

Supplementary Figure S1. Normalized absorbance spectra of CAISWG (green dot line), QISWG (blue short dash), ARWG (red solid line) and SRWG (black dash line).

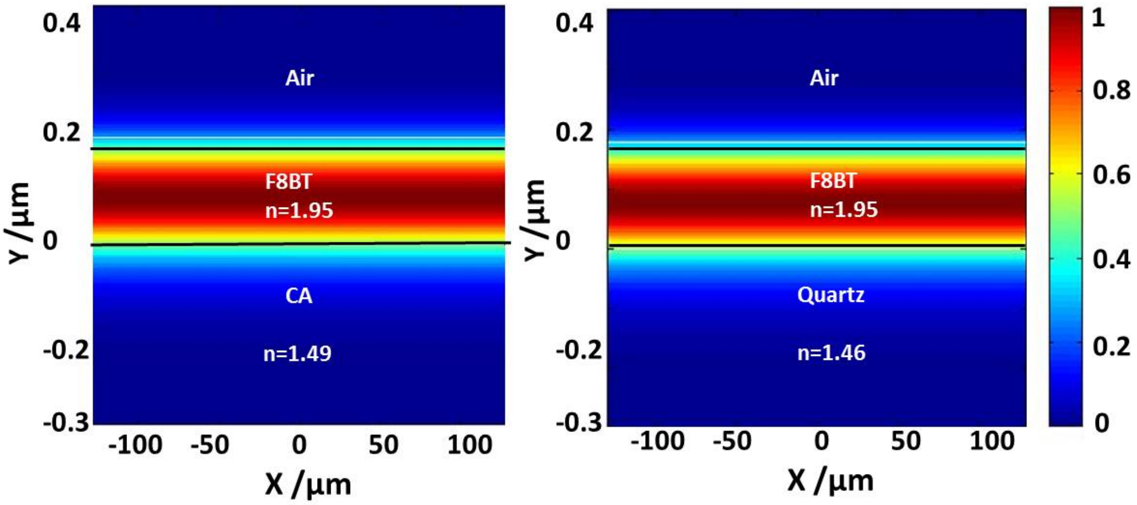

Supplementary Figure S2. Simulation of TE_{00} waveguide mode profiles at the wavelength of 570 nm in a) QISWG and b) CAISWG.

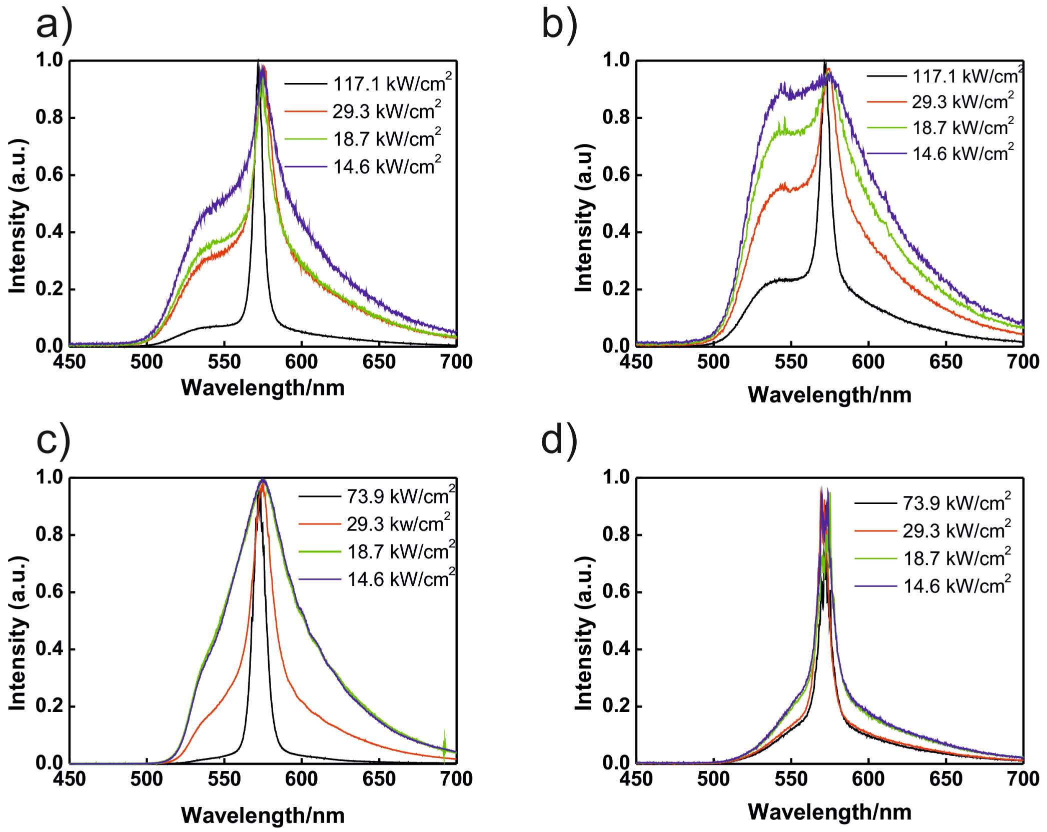

Supplementary Figure S3. Normalized output spectra at different pump power densities of a) QISWG, b) CAISWG c) ARWG and d) SRWG.

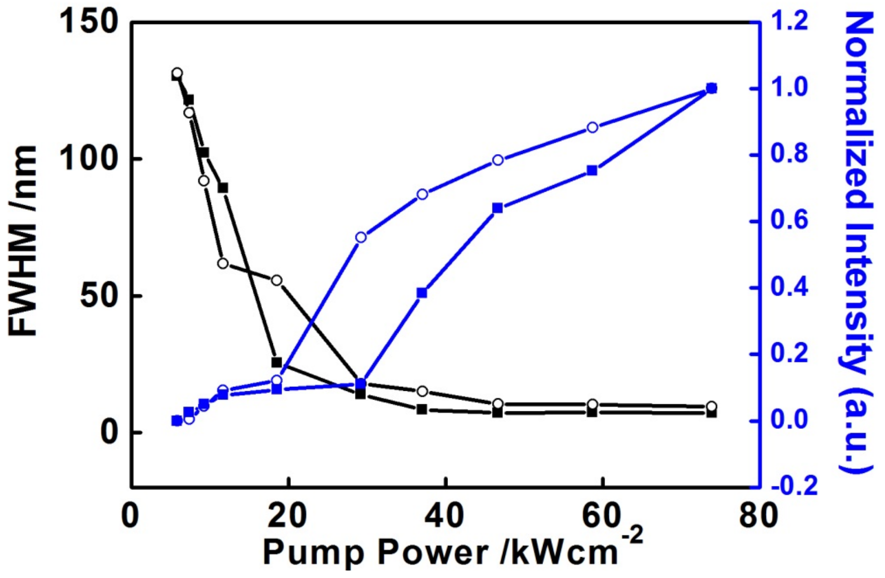

Supplementary Figure S4. Full widths at half-maximum (FWHM) and their light emission peak intensities of QISWG (black and blue squares respectively) and ARWG (black and blue circles respectively), as a function of the incident laser fluence.

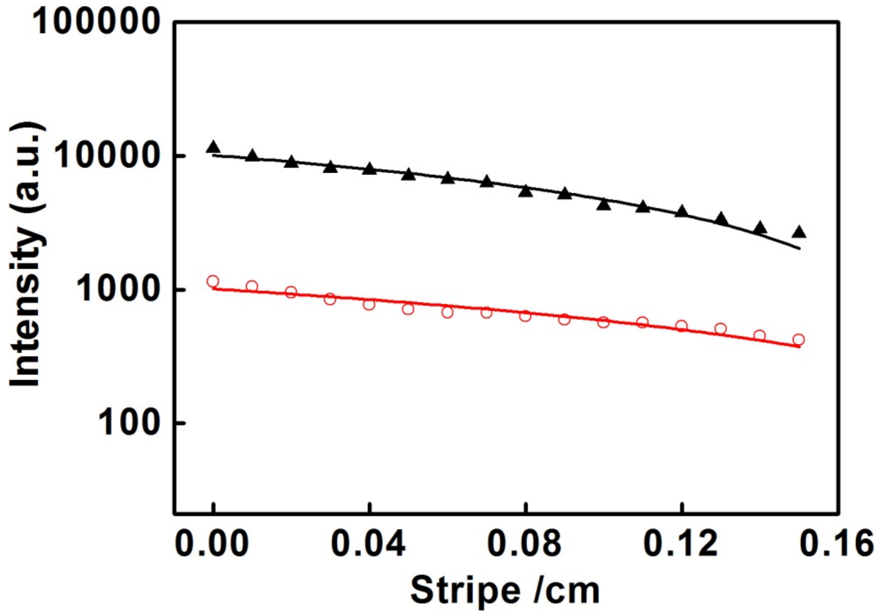

Supplementary Figure S5. Dependence of the emission intensity at λ_{ASE} on the length of the unpumped region between the edge of the excitation stripe and the substrate edge of CAISWG (black triangles) and QISWG (red circles). Black solid line and red solid line represent a linear fit of each data set.

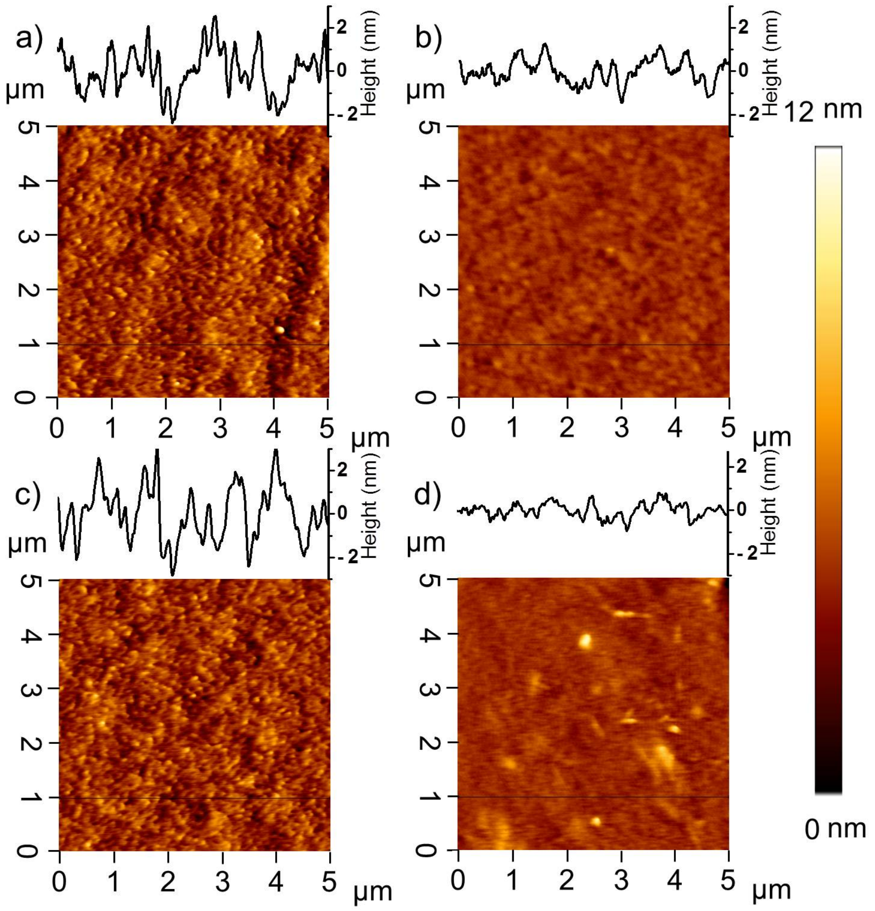

Supplementary Figure S6. AFM topography images of a) CAISWG (RMS=1.2 nm), b) QISWG (RMS=0.5 nm), c) ARWG (RMS=1.0 nm), d) SRWG (RMS=0.7 nm). Respective profiles are included above each image. The corresponding upper layer surfaces measured were F8BT for CAISWG, QISWG and ARWG and the CA solution processed layer in the case of SRWG.

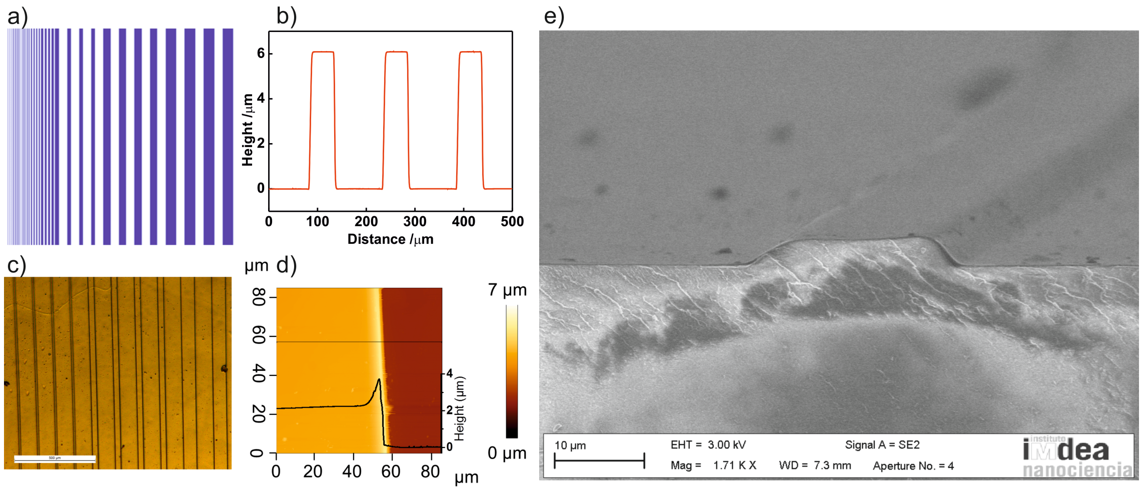

Supplementary Figure S7. a) 2D design of the Si ridge waveguide master mold, with different widths of 5, 10, 20, 50, 100 and 200 µm with four repetitions for each width. The waveguide depth is 6 µm. b) Profile measurement of the PDMS mold. c) Optical microscope image of the surface of the ridge waveguide microstructure printed in a CA substrate. The defects that appear in the surface in the form of comets are due to aggregates that are characteristically observed in films deposited by spin coating. Scale bar is 500 µm. d) Step height of the ARWG measured by AFM. e) FESEM cross-section image of an ARWG sample. Measurement conditions are included in the image. In order to improve the contrast of the image a 6 nm Au coating was sputtered onto the surface.
